# AGEs Induced Autophagy Impairs Cutaneous Wound Healing via Stimulating Macrophage Polarization to M1 in Diabetes

**DOI:** 10.1038/srep36416

**Published:** 2016-11-02

**Authors:** Yuanyuan Guo, Cai Lin, Peng Xu, Shan Wu, Xiujun Fu, Weidong Xia, Min Yao

**Affiliations:** 1 Department of Burns and Plastic Surgery, Shanghai Ninth People’s Hospital, Institute of Traumatic Medicine; Shanghai Jiao Tong University School of Medicine, Shanghai, 201900, China; 2 Burn and Wound center, First Affiliated Hospital of Wenzhou Medical University, Wenzhou, 325000, China; 3 Wellman Center for Photomedicine, Massachusetts General Hospital, Harvard Medical School, Boston, MA 02114, USA

## Abstract

Autophagy is essential in physiological and pathological processes, however, the role of autophagy in cutaneous wound healing and the underlying molecular mechanism remain elusive. We hypothesized that autophagy plays an important role in regulating wound healing. Here, we show that enhanced autophagy negatively impacts on normal cutaneous healing process and is related to chronic wounds as demonstrated by the increased LC3 in diabetic mice skin or patients’ chronic wounds. In addition, inhibition of autophagy by 3-MA restores delayed healing in C57BL/6 or db/db mice, demonstrating that autophagy is involved in regulating wound healing. Furthermore, we identify that macrophage is a major cell type underwent autophagy in wounds and increased autophagy induces macrophages polarization into M1 with elevated CD11c population and gene expressions of proinflammatory cytokines. To explore the mechanism underlying autophagy-impaired wound healing, we tested the role of IRF8, a regulator of autophagy, in autophagy-modulated macrophages polarization. IRF8 activation is up-regulating autophagy and M1 polarization of macrophages after AGEs (advanced glycation endproducts) treatment, blocking the IRF8 with shIRF8 inhibits autophagic activity and M1 polarization. In summary, this study elucidates that AGEs induces autophagy and modulates macrophage polarization to M1 via IRF8 activation in impairment of cutaneous wound healing.

Wound healing is a complex and dynamic process for restoration of injured cellular structures and tissues, traditionally explained in terms of three overlapping phases: inflammation, proliferation, and maturation^1^. There are a number of factors (age, health status, nutrition, infection, stress, medication, and others) that can affect this process, and cause impaired wound healing. In general terms, delayed acute wounds and chronic wounds are considered as two main types of impaired healing. Wounds that heal faster are classified as acute wounds; chronic wounds are classified as wounds with prolonged healing time. The normal repair process can be interrupted at any phase and is vulnerable to a variety of inhibitory factors^2^. It is noteworthy that an immunosuppressive drug of sirolimus/rapamycin for anti-rejection of grafting, as an inducer of autophagy, has been implicated in impeded healing in patient and rats studies^3^,^4^,^5^, suggesting autophagy is associated with wound healing. In addition, diabetes, as a significant factor, can affect healing process and lead to chronic wounds.

Diabetes mellitus (DM) is one of the most common metabolic diseases worldwide, the morbidity and mortality of which are increasing annually. Diabetic patients are much more susceptible to developing chronic wounds, especially on the foot. Diabetic foot ulcers are considered one of the most serious complications of diabetes, representing a major medical problem that affects quality of life and precedes 84% of diabetes-related lower extremity amputations^6^. The chronic wounds that diabetic patients experience are related to inadequate circulation, poorly function of veins, dysfunction of cells, and neuropathy^7^,^8^. The formation of advanced glycation endproducts (AGEs), a group of metabolic heterogeneous compounds, and the interaction with their receptors are also responsible for delayed or impaired repair in individuals with diabetes^9^. Despite considerable research focused on understanding and treating of diabetic wounds, the molecular mechanisms underlying impaired diabetic wounds are poorly understood. In addition, there are no studies that have investigated the role of autophagy in diabetic impaired wound progression.

Autophagy, the process by which cells recycle cytoplasm and dispose of excess or defective organelles, plays an essential role for survival, differentiation, and homeostasis^10^,^11^,^12^. Autophagy not only contributes to human physiological events, but also can paradoxically cause some of pathological conditions^13^. Recently, there has been increased evidence that autophagic dysfunction could be implicated in the development of neuro-degenerative diseases, cancer, infection and aging diseases^10^,^11^. Impaired autophagy in the liver of Atg7 deficient mice leaded to disorganized hepatic lobules and cell swelling by accumulation of abnormal organelles, which exerted cytotoxic effects on hepatocytes^14^. While autophagy enhancement through over-expression of Atg1 can cause striking inhibition of cell growth and even result in apoptotic cell death in drosophila^15^. Furthermore, autophagy is critical for regulating inflammatory reaction in innate and adaptive immune response of various organs, including intestine, lung, and kidney^16^,^17^,^18^. However, it is unclear whether autophagy acts primarily on inflammatory response in skin, during cutaneous wound closure.

Inflammatory response is a fundamental type of response by body tissue to harmful stimuli, such as disease and injury, which is indispensable in wound healing^19^. As a prominent inflammatory cell in wounds, macrophage derived from circulating monocyte precursor after injury exerts coordinating inflammation and angiogenesis phases during wound healing^20^. Macrophage polarization refers to development of two different specific phenotypes (M1 & M2) in respond to local environmental stimuli^21^. Classically activated macrophages (M1) promote inflammation in early stage of wound healing, but alternatively activated macrophages (M2) exhibit an anti-inflammatory function in lately stage of the process^20^. Autophagy has been recognized as important in macrophage polarization very recently. It has been demonstrated that TFEB (transcription factor EB) activation increased gene expression of autophagy and caused rapidly induction of M1-related proinflammatory cytokines and chemokines in murine macrophage^22^. Another study defined that rapamycin-induced autophagy stimulated the differentiation of monocytes into M1 macrophages and secretion of proinflammatory cytokines (IL-12, IL-6, TNF-a and IL-1) in human^23^. In contrast, there are some controversial results that autophagy inhibited BMDM (Bone Marrow-Derived Macrophage) exhibited up-regulation of M1 and down-regulation of M2 by high-fat diet in Atg5 knockout mice^24^. Thus, it still remained unclear whether autophagy modulates macrophage polarization into M1 or into M2 in cutaneous normal and impaired wound healing.

In view of the key role of macrophage polarization in normal and impaired wound healing and the importance of autophagy in inflammation regulation, we focused mainly on that the effects of autophagy on healing process and mechanisms underlying autophagy caused impaired wound healing in this study. We found that enhancement of autophagic activity negatively impacts on normal and chronic wound healing *in vivo* and that increased autophagy stimulates macrophage polarization to M1 in cells after AGEs treatment, but blockage of autophagy activity restores impaired healing and inhibits macrophage polarization to M1. In addition, we determined IRF8 up-regulates macrophage polarization to M1 in response to autophagy enhancement.

## Results

### Enhancement of autophagic activity induced by rapamycin impairs cutaneous wound healing

**Figure 1:**
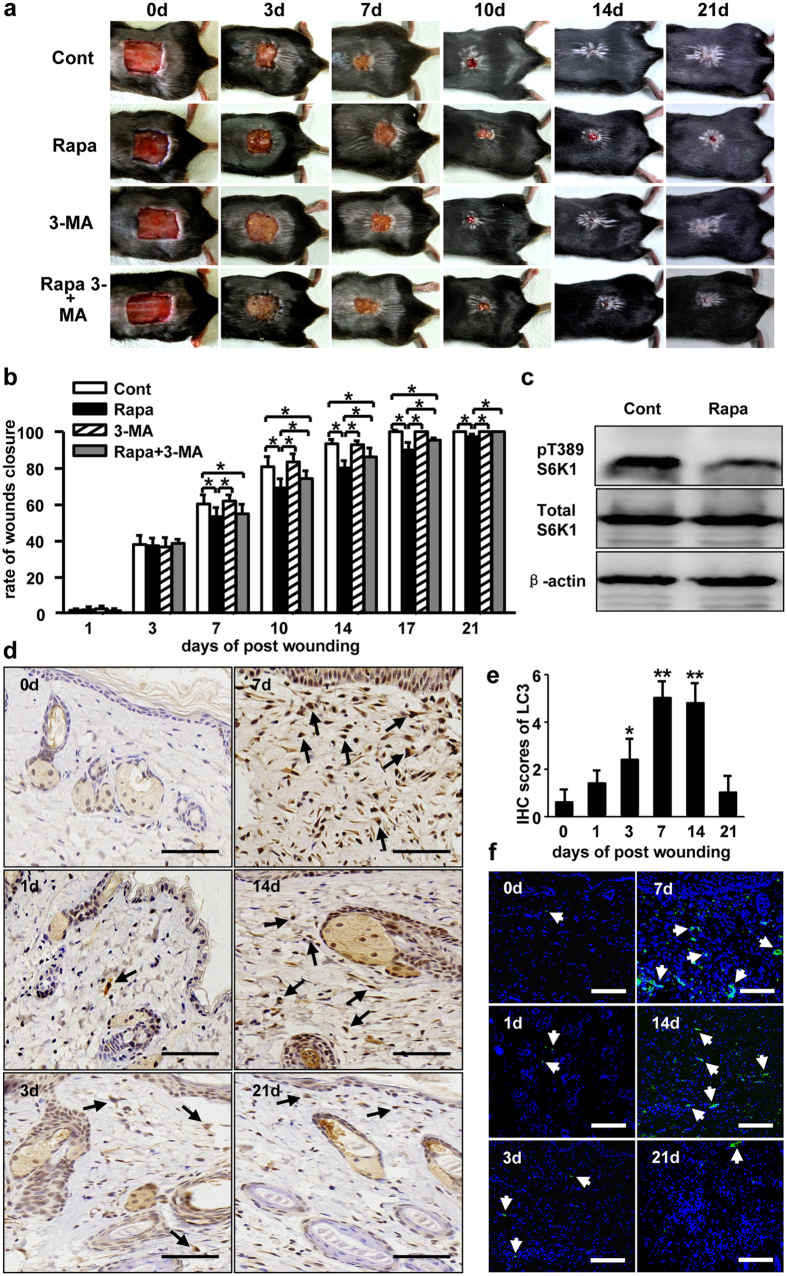
The role of autophagy in wound healing. (**a**) A full-thickness skin defect of 1.5 × 1.5 cm^2^ was made on C57BL/6 mouse. The mice were treated with rapamycin, 3-MA (10 mg/kg/d), rapamycin pluse 3-MA by i.p. Equal volume of PBS was used as control. Representative photographs of wounds closure are presented as indicated. (**b**) The areas of wounds were quantified and the ratios of wounds closure were expressed as a percentage of repaired wound compared to the area on day 0 (4 groups, n = 10 per group). Data is presented as the mean ± SD. *P < 0.05. (**c**) The levels of pT389-S6K1 in wounds with and without rapamycin (Rapa) treatment were detected by Western blot analysis on day 7 post-wounding. (**d**) Skin tissues from the control animals that wounded only were harvested for LC3 staining using IHC. Brown staining stands for the positive cells as indicated by black arrows. Scale bars = 100 μm. (**e**) The average scores of LC3 staining were evaluated using IHC (4 groups, 6 time points as indicated, n = 6 per group per time point). Data is presented as the mean ± SD. *P < 0.05, **P < 0.01. (**f**) Immunofluorescence was carried out with LC3 antibody in tissue from the control animals that wound only. White arrows indicate the positive cells with green staining. Blue staining represents nucleus. Scale bars = 100 μm.

To examine how autophagy impacts normal wound healing process, we created a murine model of full thickness skin defect in which mice were treated with rapamycin daily. A series of photographs for wound closure were presented over a period of 21 days (Fig. 1a). The mice treated with Rapamycin, which induced autophagy by blocking mTOR pathway, exhibited obviously delayed in the rate of wounds healing (22.1 ± 0.85 days) as compared to those control (treated with vehicle, 17 ± 0.94 days). The significant differences of healing rate between the control and Rapamycin treatment groups were found on days 7, 10, 14, 17 and 21 post-wounding (Fig. 1b). These data indicate that rapamycin delays normal wound repair process. Considering that rapamycin-mediated mTOR pathway inhibition (Fig. 1c) not only causes the cells to undergo autophagy, but also affects cell proliferation and metabolism through signaling cascades. We try to determine whether autophagy induced by rapamycin-mediated mTOR inhibition is the main cause for impaired wound closure. Autophagy inhibitor, 3-MA, was applied to mice by intraperitoneal injection before rapamycin treatment. Compared to that in treated with rapamycin mice alone, the rate of wound healing in the 3-MA plus rapamycin treatment mice was accelerated (19.7 ± 0.57 days). Application of 3-MA to mice rescued rapamycin-induced impaired wound healing, although not completely (Fig. 1a,b), implicating that autophagy plays an important role in rapamycin delayed wound closure.

**Figure 2:**
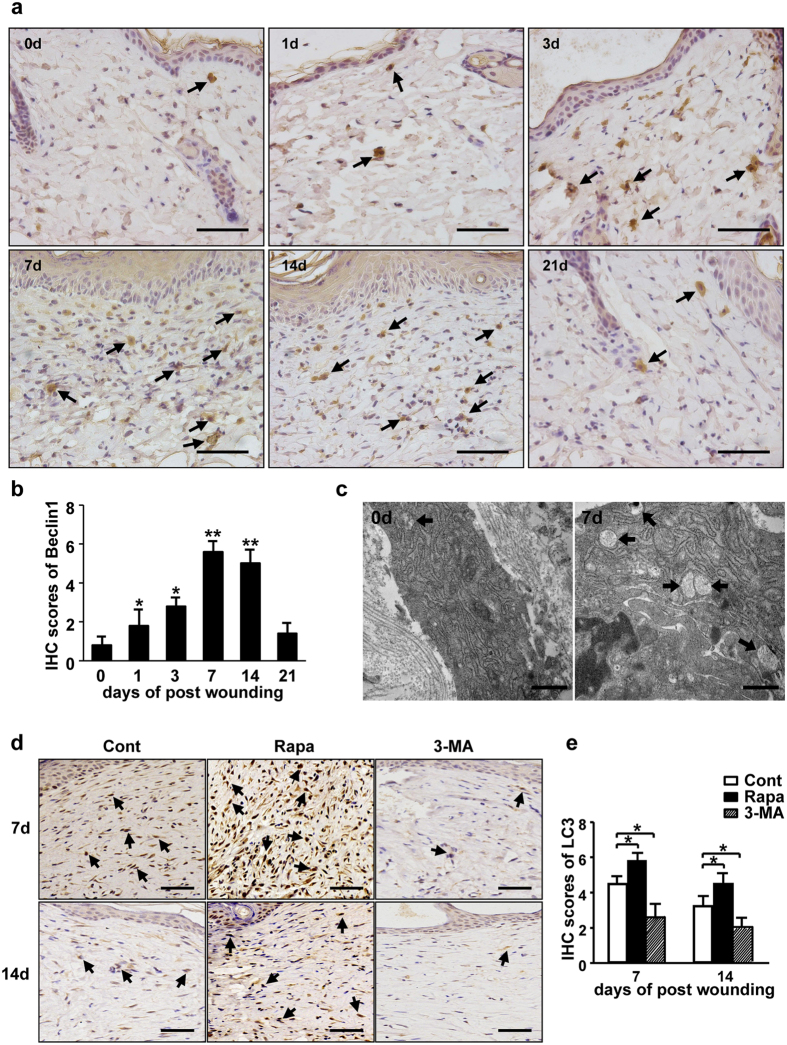
The changes of autophagy during wound healing. (**a**) Skin tissues from the control animals that wounded only were harvested for beclin-1 detection using IHC with beclin-1 antibody. The positive cells are brown as indicated by black arrows. Scale bars = 100 μm. (**b**) The average scores of beclin-1 staining were evaluated (4 groups, 6 time points as indicated, n = 6 per group per time point). Data is presented as the mean ± SD. *P < 0.05, **P < 0.01. (**c**) Electron microscopy was employed to observe numbers of autophagosome and autolysosome in wounds. Arrows indicate autophagosome or autolysosome. Scale bars = 500 nm. (**d**) The LC3 staining was sperformed in wounds with rapamycin or 3-MA treatment. Brown staining represents the positive cells as indicated by black arrows. Scale bars = 100 μm. (**e**) The average scores of LC3 staining were evaluated at different time points as indicated (n = 6 per group per time point). Data is presented as the mean ± SD. *P < 0.05.

In addition, to determine the kinetic changes of autophagy during cutaneous wound repair, we used several common methods to detect autophagic levels in skin. Firstly, the animal skin tissues were stained with two types of autophagic markers, LC3 and beclin-1, using IHC or immunofluorescence. The levels of LC3 (Fig. 1d–f) and beclin-1 (Fig. 2a,b) in the wounded mice skin began to increase on Day 1, and reach to the highest level on Day 7, and then decreased gradually to the basal level on Day 21 post-wounding. Secondly, to confirm whether the skin tissue cells experience autophagy, electron microscopy was used to monitor the number of autophagic lysosomes. A significantly increased number of autolysosome was defined in wounded mice skin on Day 7 (Fig. 2c). Furthermore, LC3 levels in the rapamycin treated mice skin were much higher than that in the control or 3-MA treated mice on Days 7 or 14 post-wounding, respectively (Fig. 2d,e). Taken together these data demonstrate that rapamycin-mediated mTOR inhibition induced autophagy has a negative impact on cutaneous wound healing *in vivo*.

### Impaired wound healing, such as diabetic wounds, is associated with increased autophagy in both mice and patients

**Figure 3:**
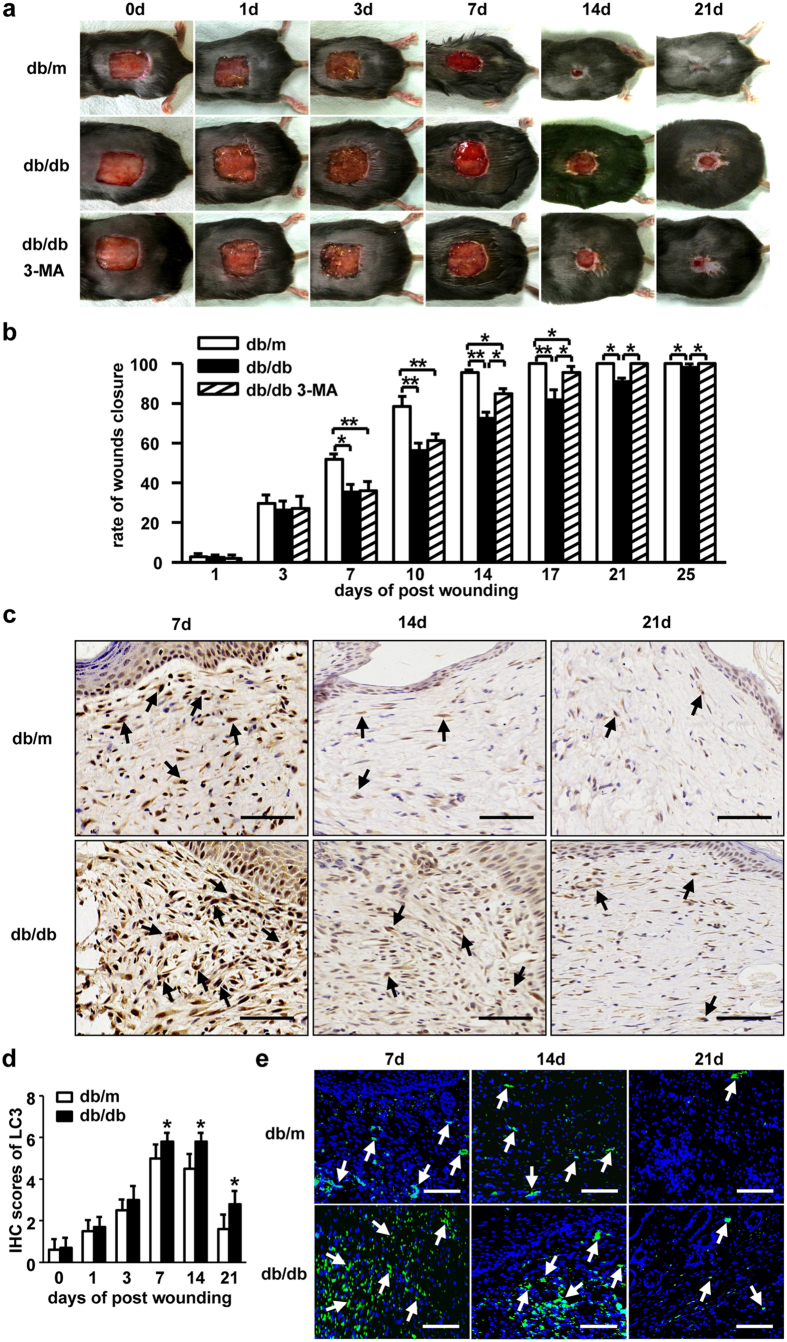
The changes of autophagy in impaired diabetic wounds healing. (**a**) A full-thickness skin defects was made on each db/db or db/m mice. The db/db mice were pre-treated with or without 3-MA (10 mg/kg/d) by i.p. from 1 day before wounding and up to the wounds closed completely. Representative photographs of wounds closure are presented. (**b**) The areas of wounds were quantified and the ratios of wounds closure were expressed (3 groups, n = 10 per group). Data is presented as the mean ± SD. *P < 0.05, **P < 0.01. (**c**) The LC3 staining was performed using IHC. The positive cells are brown as indicated by black arrows. LC3 positive cells in wounds in db/db mice are compared to that in db/m mice. Scale bars = 100 μm. (**d**) The average scores of LC3 staining were evaluated (two groups, 6 time points as indicated, n = 6 per group per time point). Data is presented as the mean ± SD. *P < 0.05. (**e**) Immunofluorescence was employed to determine LC3 positive expressions. Green staining represents the positive cells as indicated by white arrows, blue staining represents nucleus. Scale bars = 100 μm.

Given the similarity of wound closure kinetics in impaired wound healing, such as diabetic wounds, to that in the rapamycin treated mice, the contribution of autophagic level to impaired wound healing in diabetic mice model was examined. Full-thickness skin defect was established on the dorsal surface of db/db and db/m mice. The skin wounds from db/m (control) mice were closured around 16 days (15.9 ± 0.82) post-injury. However, the wounds from diabetic db/db mice were still open up to 25 days (25.8 ± 0.59) after wounding (Fig. 3a row 1 and row 2, 3b). A delay in the closure of wounds in comparison to control mice was clearly seen from Days 7 to 25. Furthermore, by using IHC or immunoflorescence staining analysis of LC3, increased autophagic levels in wounded skin from db/db mice were observed from Day 3 to 21 post-wounding as compared to that from db/m mice (Fig. 3c–e). Significantly rise of autophagic level in db/db mice were especially evident on Days 7, 14 and 21 post-wounding. The kinetics of wound closure and the patterns of autophagic level from db/db mice skin were similar to those in skin wounds from rapamycin treated C57BL/6 mice. These results indicate that increased level of autophagy is linked to impaired wound healing such as diabetic wounds.

To determine whether increased autophagy affects the diabetic wound healing process, administration of 3-MA was performed in db/db mice prior to wounding and wound closure was assessed. 3-MA treatment did significantly restore delayed wound repair in db/db mice (Fig. 3a row 2 and 3, 3b). Upon inhibition of autophagy, dysfunctional wound healing in db/db mice became restored partially on Days 14, 17, 21 and 25 after wounding. These studies demonstrate that increased autophagy is one of the major causes for dysfunction of wound healing in diabetes mice.

**Figure 4:**
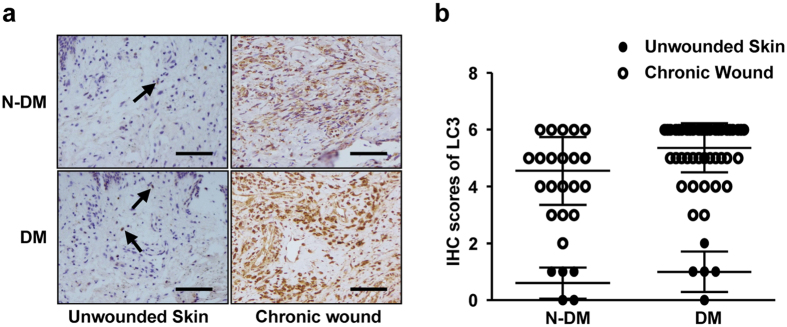
The autophagic activity in chronic wounds patients with or without diabetes. (**a**) The positive stainings of LC3 were detected in the unwounded skins and chronic wounds from patients with or without diabetes using IHC. The brown staining represents positive cells as indicated by black arrows. Scale bars = 100 μm. (**b**) Scatterplots for the average scores of LC3 staining are presented.

Since our data has shown that increased autophagy has a negative impact on both normal and diabetic wound healing in mice, it would be intriguing to determine whether it is possible to happen in patients. In an effort to verify our findings in animal experiments, we examined the autophagic level in patient tissue, including chronic cutaneous wounds with or without diabetes and unwounded skin, by using LC3 immunostaining assay. As the data shown (Fig. 4a,b), the autophagic levels in the chronic cutaneous wounds, no matter with or without diabetes, were significantly higher than that in the unwounded skin. Intriguingly, an increase of autophagy in the patient wounds with diabetes (*p* *<* 0.05) was dramatic, compared to that in the patient wounds without diabetes (Table 1). These results further confirm that increased autophagy affected cutaneous wound healing negatively in both mice and patients.

### AGEs play a critical role in increased autophagy and impaired wound healing *in vivo*

**Figure 5:**
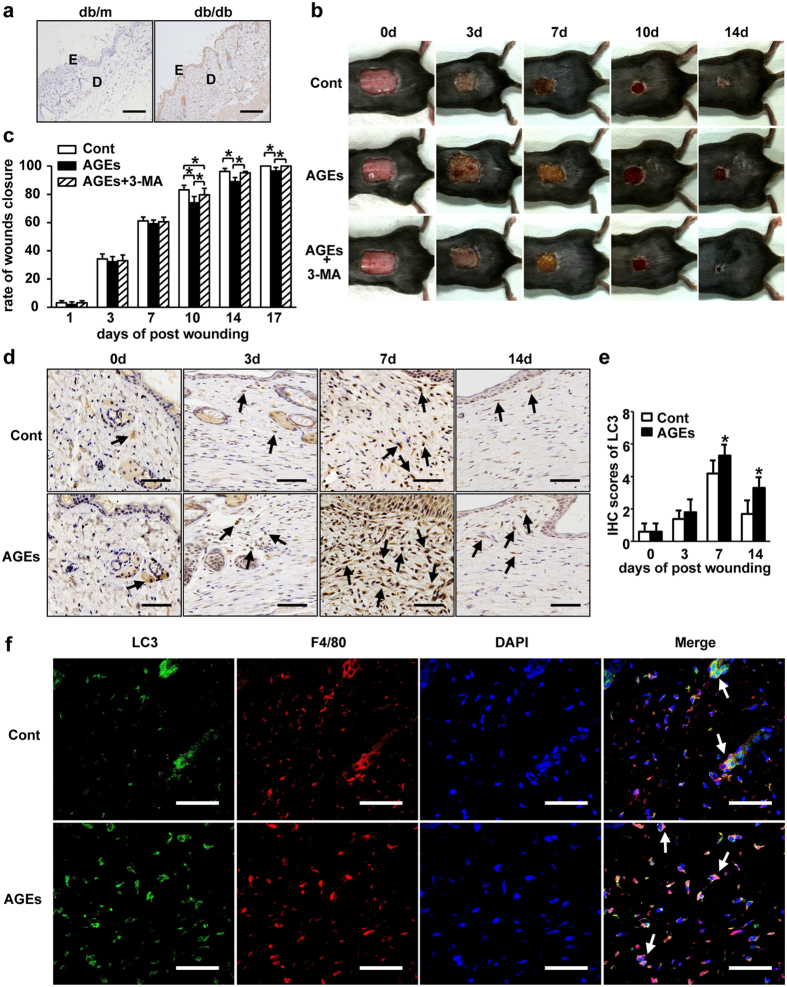
The effects of AGEs on autophagy in delayed diabetic wound healing *in vivo*. (**a**) IHC was performed for AGEs staining in skin tissues from db/db and db/m mice. The brown staining stands for positive expression. E and D indicated epidermis and dermis, respectively. Scale bars = 100 μm. (**b**) A full-thickness skin defects was made on each C57BL/6 mouse. AGEs (100 mg/kg/d) were injected into margin tissues of wound immediately after wounding with and without 3-MA pre-treatment from 1 day before wounding, up to the wounds closed completely. Equal volume of PBS was used as control. Representative photographs of wounds closure are presented. (**c**) The areas of wounds were quantified at different time points as indicated and the ratios of wounds closure are expressed (three groups, n = 10 per group per time point). Data is presented as the mean ± SD. *P < 0.05. (**d**) IHC was carried out in tissue sections with LC3 antibody. The brown staining represents positive cells as indicated by black arrows. Scale bars = 100 μm. (**e**) The average staining scores of LC3 were evaluated (two groups, 4 time points as indicated, n = 6 per group per time point). Data is presented as the mean ± SD. *P < 0.05. (**f**) Immunoreactivity of LC3 (green) and macrophage marker F4/80 (red) in wounds on days 7 post-wounding were visualized by double staining with Alexa 488 and Alexa 594. Images were taken and analyzed under Laser Scanning Confocal Microscope. Scale bars = 50 μm.

In order to determine the main factor that causes impaired wound healing and increased autophagy in diabetes. AGEs was selected as a candidate to study. AGEs, well-known harmful compounds formed by glycation reaction, which is believed to play a causative role in the development and worsening of many diseases such as diabetes mellitus, was selected to study. AGEs positive staining was observed in db/db mice skin, however, non-staining was found in the skin from db/m mice (Fig. 5a). To determine whether AGEs is able to affect on dysfunctional healing via increasing autophagy, we used AGEs to treat mice wounds by injection. Treatment with AGEs apparently delayed wounds closure (18.0 ± 0.67 vs 14.8 ± 0.79 days) in mice skin. Furthermore, administration of 3-MA completely rescued the AGEs delayed wound healing (15.0 ± 0.82 days) in C57BL/6 mice (Fig. 5b,c). In addition, LC3 positive cells in the AGEs treated mice skin significantly increased on Days 7 and 14 post-wounding, compared to those in the control group (Fig. 5d,e). These data demonstrate that AGEs was a stimulator for autophagic activity and caused impaired wound healing.

### AGEs induced autophagy negatively impacts wound healing via stimulating macrophage polarization to M1 in diabetes

**Figure 6:**
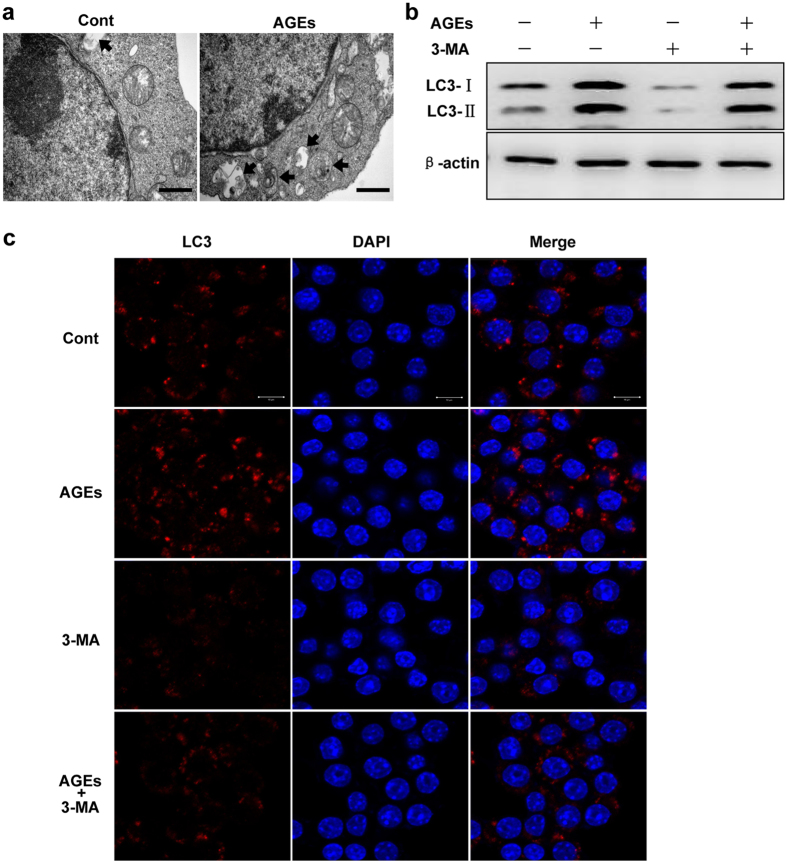
The effects of AGEs on macrophages. (**a**) RAW264.7 cells were treated with AGEs (100 μg/ml) for 24 h, numbers of autophagosome and autolysosome were observed using electron microscopy. Scales bar = 500 nm. (**b**) RAW264.7 cells were treated with AGEs, 3-MA (2 mM), and AGEs plus 3-MA for 24 h, respectively. CQ (50 nM) was added simultaneously for 24 h to inhibit autophagic flux. LC3 protein bands were obtained using western blotting. (**c**) After treating with AGEs, 3-MA, or AGEs plus 3-MA for 24 h, the cells were stained with LC3 antibody using immunofluorescence. Representative photographs were taken under Laser Scanning Confocal Microscope. The LC3 positive cells (red) are presented, blue staining represents nucleus. Scale bars = 10 μm.

**Figure 7:**
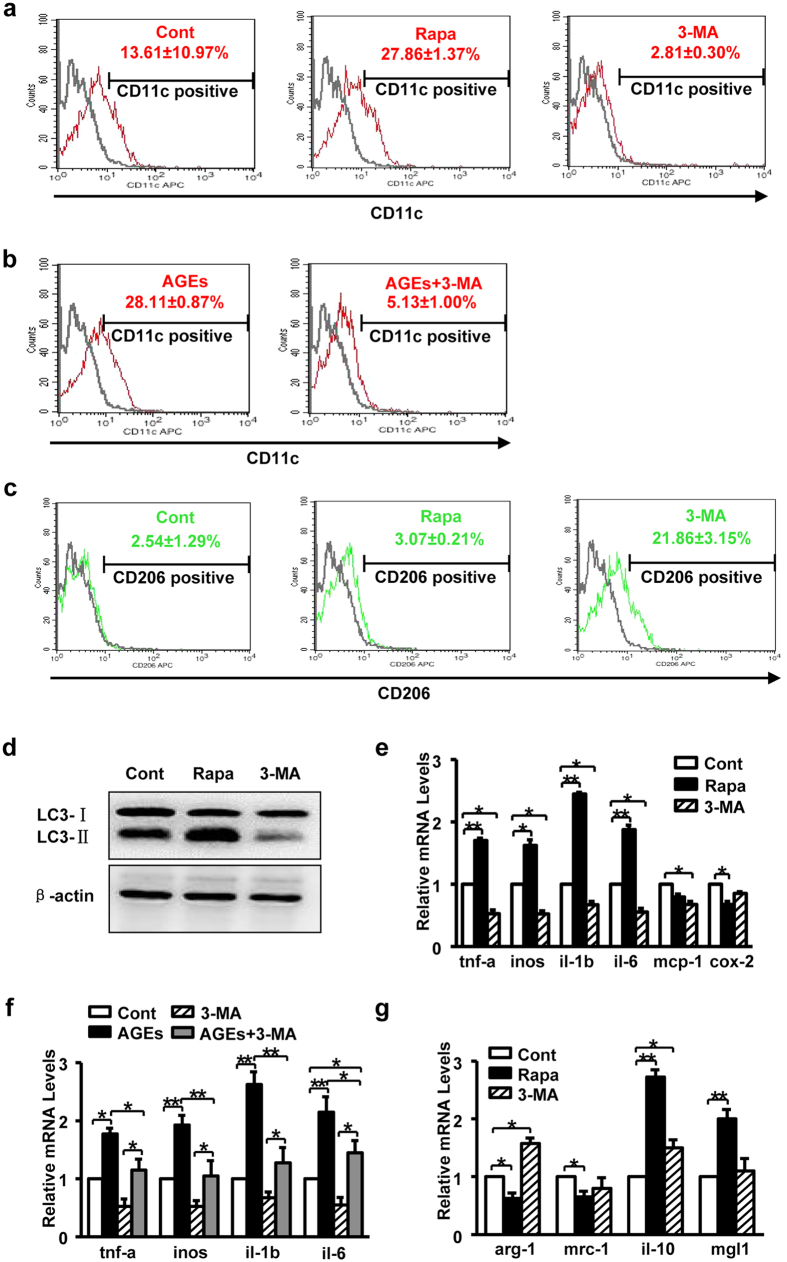
Impacts of rapamycin and AGEs on macrophage polarization. (**a**) RAW264.7 cells were treated with rapamycin (100 nM) or 3-MA for 24 h, LPS (100 ng/ml) was used simultaneously for 24 h to stimulate macrophage polarization to M1. CD11c (M1 marker) positive cells were analyzed by flow cytometry (n = 2). (**b**) RAW264.7 cells were treated with AGEs, AGEs plus 3-MA for 24 h, respectively. LPS was used for M1 polarization. CD11c (M1 marker) positive cells were analyzed by flow cytometry (n = 2). (**c**) After treatment with rapamycin or 3-MA for 24 h, IL-4 (10 ng/ml) was used simultaneously for 24 h to stimulate macrophage polarization to M2. CD206 (M2 marker) positive cells were analyzed by flow cytometry (n = 2). (**d**) RAW264.7 cells were incubated with rapamycin or 3-MA, and CQ was added simultaneously for inhibiting autophagic flux. After 24 h, western blotting was performed with LC3 and β-actin antibody. (**e**) RAW264.7 macrophage cells were treated with rapamycin or 3-MA for 24 h, respectively. LPS was used for M1 polarization. Relative mRNA levels of M1 related markers (*tnf-a, inos, il-1b, il-6, mcp-1* and *cox-2*) were detected by real-time PCR (n = 3). Data are presented as the mean ± SD.*P < 0.05, **P < 0.01. (**f**) RAW264.7 cells were treated with AGEs, 3-MA, and AGEs plus 3-MA for 24 h, respectively. LPS was added simultaneously to stimulate M1 polarization. Relative mRNA levels of *tnf-a, inos, il-1b,* and *il-6* were detected by real-time PCR (n = 3). Data are presented as the mean ± SD.*P < 0.05, **P < 0.01. (**g**) Relative mRNA levels of M2 related markers (*arg-1, mrc-1, il-10* and *mgl1*) were detected in RAW264.7 cells after treatment with rapamycin or 3-MA plus IL-4 for 24 h. IL-4 was used for stimulating M2 polarization (n = 3). Data are presented as the mean ± SD.*P < 0.05, **P < 0.01.

To elucidate molecular mechanisms underlying autophagy caused impaired wound healing in diabetes, we focused on the cell type happened autophagy after AGEs stimulation *in vivo*. Multiple-immunofluorescence staining test was utilized for identification of autophagic cell and cell type with different antibodies, including autophagy marker LC3 (green), and macrophage marker F4/80 (red). The staining results of wounded skin from both control and AGEs treated mice revealed that the most of autophagic cells in the dermis were F4/80 positive cells (Fig. 5f), suggesting macrophages are major cells undergo autophagy during wound healing. Next, we used the macrophage cell line RAW264.7 cells for detecting autophagy in *vitro*. RAW264.7 cells were treated with AGEs. Following, autophagolysosome and LC-3 protein level were examined. As shown in Fig. 6, AGEs treated cells contained more autophagolysosome compared to cells treated by vehicle (Fig. 6a) under electronic microscopy. In addition, LC3-II isoform also increased by AGEs treatment (Fig. 6b). Coapplication of AGEs and 3-MA significantly inhibited proteins of LC3 in both western blotting and immunofluorescence results (Fig. 6b,c). Furthermore, rapamycin treated RAW264.7 cells shown consistent results with AGEs treated cells (Fig. 7d). Our data suggests AGEs induce autophagy in macrophage cells.

It has been reported that macrophage is essential for wound healing by coordinating inflammation and angiogenesis. Recently, classification of macrophage recognizes polarization into two distinct phenotypes, named as proinflammatory (M1) or anti-inflammatory (M2). Polarization of macrophage to M1 or M2 regulates wounds repair. Therefore, we further identified whether AGEs or rapamycin induce macrophage polarization via stimulating autophagic activity. Polarization of macrophage to M1/M2 was determined by CD11c or CD206 and related markers. Our results exhibited that the numbers of CD11c (M1 marker) positive cells in rapamycin treated group (27.86 ± 1.37%) were much higher than that in control (13.61 ± 10.97%). By pre-treatment of 3-MA, the numbers of positive cells were diminished (2.81 ± 0.30%) (Fig. 7a). Unexpected, there was no significant difference between control and rapamycin treated groups in positive cells of CD206 (M2 marker), however, 3-MA treated cells (21.86 ± 3.15%) were prone to M2 polarization (Fig. 7c).

To confirm the flow cytometry data, we have also used Real-time PCR for identifying gene expressions of M1/M2 related markers. Gene expressions of M1 related proinflammatory mediators including *tnf-a, inos, il-1β* and *il-6,* obviously increased after treatment with rapamycin. However, pre-treatment with 3-MA inhibited the gene expressions of M1 related markers (Fig. 7e). Meanwhile, the gene expressions of M2 markers, including *arg-1, mrc-1, il-10* and *mgl1*, had no accordant changes to autophagic activity (Fig. 7g). These data indicate that increased autophagy is paralleled by macrophage polarization to M1. Next, macrophages were treated with AGEs, and then polarization was assessed. As results shown, AGEs also increased the numbers of CD11c positive cells (28.11 ± 0.87%) and the gene expressions of M1 related factors, which indicating AGEs induce macrophage polarization to M1. Meanwhile, CD11c positive cells that induced by AGEs were also decreased by 3-MA (5.13 ± 1.00%), the changes of the gene expressions of M1 related markers were consist to the data by flow cytometry (Fig. 7b,f). Taken together these results illustration that AGEs stimulated autophagic activity negatively impacts diabetic wound healing through inducing macrophage polarization to M1.

### IRF8 is involved in AGEs-induced macrophage autophagy and M1 polarization

**Figure 8:**
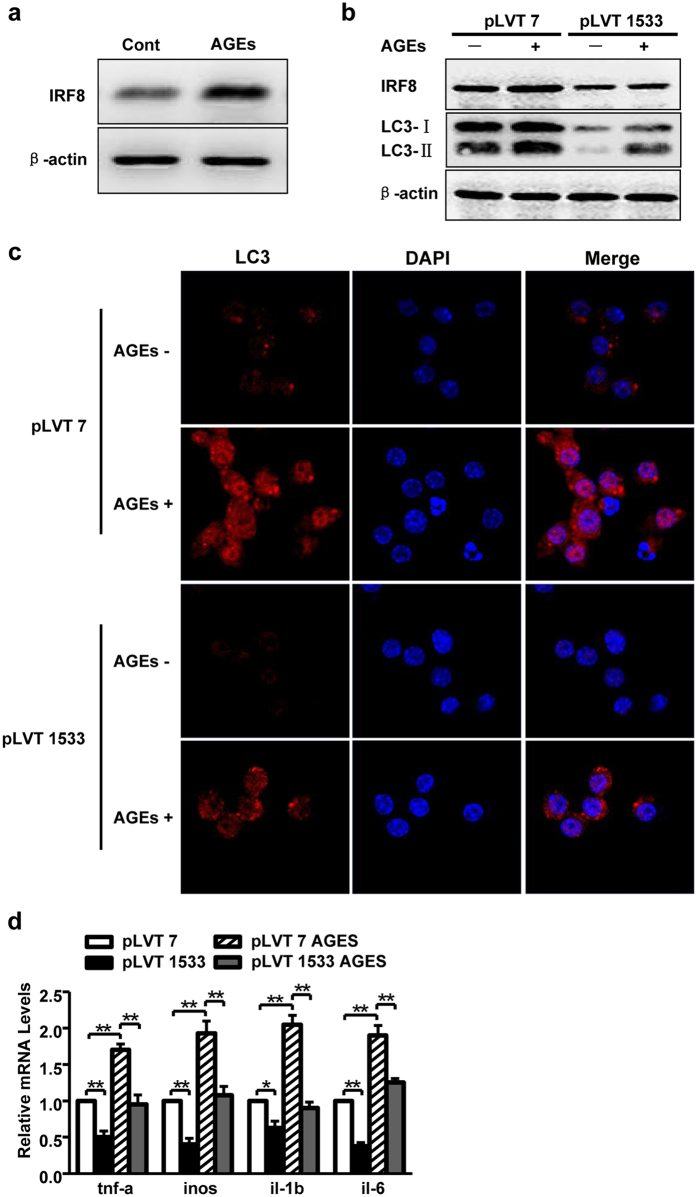
The role of IRF8 in AGEs induced autophagy and macrophage polarization. (**a**) The effect of AGEs on IRF8 was analyzed. The RAW264.7 cells were treated with or without AGEs for 24 h, CQ (50 nM) was added simultaneously for 24 h to inhibit autophagic flux. Western blotting was performed to demonstrate IRF8 protein bands. (**b**) The RAW264.7 cells, infected with pLVT 7 (control shRNA) or pLVT 1533 (shIRF8), were treated with and without AGEs (100 μg/ml) for 24 h. Western blotting was performed to demonstrate IRF8 and LC3 protein bands. (**c**) The infected cells were incubated simultaneously with AGEs for 24 h, immunofluorescence was employed for LC3 staining. The positive cells (red) are presented, blue staining represents nucleus. (**d**) The infected cells were stimulated by LPS for M1 polarization with and without AGEs treatment for 24 h. Relative mRNA levels were detected using real-time PCR (n = 3). Data is presented as the mean ± SD.*P < 0.05, **P < 0.01.

Recent study demonstrates that IRF8 is a major regulator for autophagy and macrophage polarization. Therefore, we try to determine whether IRF8 is involving in AGEs-induced macrophage autophagy and M1 polarization. RAW 264.7 cells were treated with AGEs and then subjected to western blotting. Administration of AGEs resulted in a 2 fold increase of protein level of IRF8 (Fig. 8a) and an elevation of autophagic activity include formation of autophagolysosome and raised proteins of LC3 (Fig. 6a–c) in macrophages. Those results indicate that IRF8 and autophagy are downstream of AGEs stimulation. In addition, in light of the importance of IRF8 in stress-activated autophagy in Ozato’s study^25^, it is tempting to postulate that IRF8 is regulating AGEs-induced autophagy. To investigate the involvement of IRF8 in our study, we designed an IRF8-specific shRNA that could reduce the IRF8 protein level of 70%. By blocking IRF8 with the shIRF8, baseline of LC3 and enhanced LC3 by AGEs were suppressed (Fig. 8b,c), suggesting IRF8 is regulated to AGEs-induced autophagy. Furthermore, the molercular pathway of IRF8 and AGEs-induced macrophage polarization was studied. As shown in Fig. 8d, gene expressions of M1 markers (*tnf-a, inos, il-1β, il-6*) in RAW 264.7 cells with and without AGEs treatment were significantly reduced after infecting with shIRF8, compared to that in the cells infected with control shRNA. These data indicate that the molecular mechanism of IRF8-autophagy-M1 polarization of macrophages plays important role in AGEs caused impairment of healing.

## Discussion

By eliminating intracellular proteins and damaged organelles, autophagy is an evolutionarily conserved degradation process for homeostasis and has emerged as a potent clinically relevant modulator for disease progression^10^. However, little is known about the modulation role of autophagy in wound healing and the underlying molecular basis. The major finding in current study is that enhancement of autophagy negatively impacts on normal wound healing or diabetic wounds through modulating macrophage polarization to M1, which is supported by the following: (1) enhanced autophagy by rapamycin delays normal skin wound healing in C57BL/6 mice and inhibition of autophagy by 3-MA restores rapamycin-delayed healing; (2) autophagic activity in the tissues from chronic wounds in diabetic mice and patients with or with out diabetes have an identical increased levels of LC3, while inhibition of autophagy by 3-MA remarkably accelerated impaired wound healing in db/db mice; (3) AGEs, harmful compounds of diabetes, induce autophagy and led to impaired wound healing and macrophage is identified as a major cell type that underwent autophagy in wounded site after administration of AGEs *in vivo*; (4) *in vitro,* increased autophagy modulates macrophages polarization into M1 with elevated CD11c population and gene expressions of proinflammatory cytokines including *tnf-a, inos, il-1β, and il-6,* which was induced by rapamycin or AGEs; (5) IRF8 is a regulator in AGEs induced autophagy, which modulated M1 macrophage polarization during wound healing. These finding suggest that enhanced autophagy by AGEs involved in impairment of cutaneous wound healing through up-regulating IRF8-autophagy-M1 polarization of macrophages.

Even autophagy has been largely linked to various diseases including cancer, heart disease, and neurodegeneration, but there is no enough evidence to show how autophagy impacts on acute and chronic wound healing in skin. In current study, we used several approaches and different wound healing models (mice and patients) to examine those questions. Here, our study ascertains that the impairment of acute healing was caused by elevated autophagic activity in C57BL/6 mice. The chronic lesion tissues from diabetic mice as well as patients with or without diabetes, typical impaired wound healing models, also appeared to be in an increased level of autophagy accompanying with raised LC3 and beclin-1 proteins. Thus, we reveal for the first time that autophagy is excessive in diabetic skin wounds, which is associated with delayed wound healing. Furthermore, specific inhibitor of 3-MA for autophagy resulted in restoration of the impeded cutaneous healing. These data verify that functional autophagic activity is involved in the modulation of skin wound healing. As we know, autophagy is active at basal levels in most cell types to play a housekeeping role in maintaining normal cell functions. However, the lossy or excessive autophagy also has the capacity to cause abnormal conditions. It has been reported that insufficient podocyte autophagy was involved in the pathogenesis of podocytes loss and led to massive proteinuria by high-fat diet in Atg5 deficient mice^26^. Contradictory excessive autophagy in adipocytes caused insulin resistance of adipose tissue in obese patients with type 2 diabetes^27^. Intriguingly, several clinic studies have reported that the major side effect for applications of sirolimus, which is an immunesuppressive agent for anti-rejection of graft after surgery, was delaying wound closure^3^,^4^. One of the possibilities is that the drug activated autophagic pathway activity since sirolimus is a specific inhibitor of mTOR and autophagy inducer. Those studies are consistent with ours, which strongly support our findings.

Among the divergent pathways and functions elicited by autophagy, the involvement of autophagy pathway in inflammation has been studied. A proper inflammatory response is required for skin wound repair. Too little inflammation could lead to tissue unhealed^28^. On the other hand, prolonged inflammation leads to pathological healing such as scarring^29^. Macrophages are the most functionally diverse cells for generation and resolution of inflammation by phenotype polarization. Polarized macrophages are classified in two distinct sub-populations: classically activated Macrophages (M1), which secrete high levels of pro-inflammatory cytokines (IL-1β, IL-6, iNOS, TNF-α) and exhibit promoting inflammatory properties, and alternatively (M2) activated Macrophages that are characterized by high IL-10 and TGF-β and low IL-12 production^20^. M2 macrophages play a role in resolution of inflammation, accompanied by reduced pro-inflammatory cytokine secretion, but an exception is the M2b^30^. Thus, autophagy may modulate inflammation through inducing macrophages polarization during wound healing. In fact, autophagy has been linked to macrophages polarization. Here we showed that autophagy induced by AGEs or rapamycin stimulated macrophage polarization to M1 with gene expressions of *il-1β, il-6, inos, tnf-α* and high population of CD11c cells, but no changes of M2 (Fig. 7). In contrast, autophagy inhibitor reduced M1 polarized macrophages. Our findings elucidate that the outcome of delayed healing was due to autophagy caused high population of M1 and sustained inflammation in mice and patients. Similarly to our findings, another study has also reported that rapamycin stimulated the monocytes to differentiate into M1 macrophages releasing more IL-12 and less IL-10 in human peripheral monocytes, whereas activated mTOR by TSC2 (the mTOR repressor tuberous sclerosis complex 2) knockdown caused M2 polarization^23^. In addition, impaired autophagy increased proinflammatory M1 and decreased anti-inflammatory M2 polarization in BMDM and Kupffer cells from Atg5 knockout mice^24^. Those discrepancies may due to various cell types and different pathological situations.

AGEs *in vivo* are modifications of proteins, lipids, or DNA mainly via the Maillard reaction that become nonenzymatically glycated and oxidized after contact with aldose sugars. Increased levels of AGEs have been implicated in several chronic diseases, including diabetes-related complications such as renal diseases, retinopathy, neuropathy, and cardiovascular diseases, as well as delayed wound healing. However, there was unclear whether AGEs is participating in regulation autophagy during wound healing. AGEs act via their receptor, RAGE, and are implicated in chronic diabetic wounds and inflammation^9^. It has been shown that chronic subclinical inflammation with high level of C-reactive protein may be partly due to increased serum concentration of AGEs in patients with diabetes^31^. Similar to AGEs, advanced lipoxidation end products (ALE) are also formed as a consequence of diabetes. Synthetic ALE could increase the expression of IL-6, IL-8, MCP-1, and iNOS in induced THP-1 monocytes^32^. Furthermore, AGEs enhanced BMDM cells polarization into M1 macrophage with producing IL-6 and TNF-a proinflammatory cytokines and upregulating the expressions of CD11c and CD86 through activating RAGE/NF-κB pathway in mice^33^. In addition, AGEs has been shown to induce autophagy in some cells, such as hepatic stellate cells^34^, cardimyocytes^35^, vascular smooth muscle cells^36^, and endothelial cells^37^. Our results for the first time show that AGEs could stimulate M1 polarized macrophage by inducing autophagy. In our study, we further identify that AGEs induced autophagy and M1 polarization through activation of IRF8, which is a regulator of autophagy^25^, which contributes to delayed wound healing. Specific inhibition of IRF8 resulted in reduction of autophagy and less M1 polarization in cells and finally ameliorated wound healing in mice. More detail molecular mechanisms for IRF8 induced autophagy-M1 polarization in wound healing will be studied in the future.

**Figure 9:**
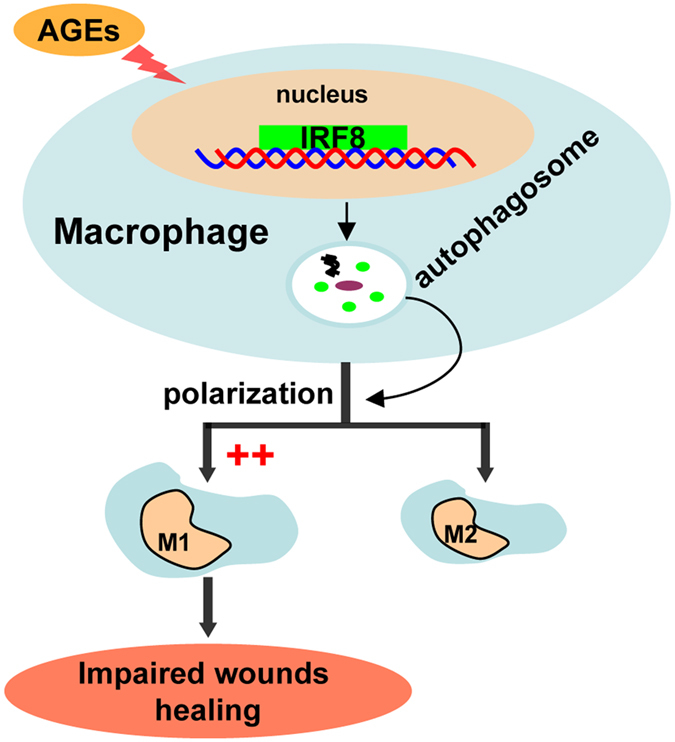
A schema for the effect of AGEs-induced autophagy on wound healing and its mechanism. AGEs up-regulate expression of IRF8 and induce macrophage autophagy. Enhanced autophagic activity promotes macrophage polarization to M1. Furthermore, increased M1 macrophages result in excessive inflammation, which leads to impaired wound healing. Together, the mechanism of IRF8-autophagy-M1 polarization is critical for AGEs-induced autophagy impaired wound healing in diabetes.

In summary, this study reveals for the first time that AGEs induces autophagy and modulates macrophage polarization to M1 via IRF8 activation in impairment of healing (Fig. 9), which suggest autophagy is essential modulator of wound healing. Targeting autophagy in therapeutic interventions may be useful for diabetic impaired wounds.

## Materials and Methods

### Materials

Rapamycin, Three-methyladenine (3-MA), lipopolysaccharide (LPS), chloroquine (CQ), penicillin, streptomycin, and bovine serum albumin (BSA) were obtained from sigma (St. Louis., MO, US). IL-4 was purchased from R&D (Minneapolis, MN, US). Lysis buffer radioimmunoprecipitation assay (RIPA), phenylmethanesulfonyl fluoride (PMSF), and bicinchoninic acid (BCA) protein assay kit were obtained from Beyotime Institute of Biotechnology (Shanghai, China). Trizol reagent and cDNA reverse transcription kit were purchased from Invitrogen (Carlsbad, CA, US). The following antibodies were purchased: anti-LC3 antibody, S6K1 and pT389-S6K1 antibodies from Cell Signaling Technology (Danvers, MA, US), anti-Beclin1 antibody, anti-F4/80 antibody, and anti-IRF8 antibody from Santa Cruz (Dallas, TX, US), anti-AGEs antibody from Abcam (Cambridge, MA, US), β-actin antibody from Bioworld Technology (Louis, MN, US), CD206-APC, and CD11c-APC for flow cytometry from BD Biosciences (San Jose, CA, US). Alexa Fluor 488 conjugated goat anti-rabbit IgG second antibodies, and Alexa Fluor 594 conjugated goat anti-rat IgG second antibodies from Thermo Scientific (Hudson, NH, US).

### AGEs preparation

AGEs were prepared by incubating BSA with 500 mmol/L of D-glucose for 16 weeks at 37 °C based on published method^38^. AGEs level was measured by clinical laboratory of No.3 hospital, Shanghai Jiaotong University School of Medicine.

### Animal model of full-thickness skin wound healing

Male mice C57BL/6 of 336 were purchased from Slac Laboratory of Animals, the total of 96 db/db mice and non-diabetic db/m mice of 48 were obtained from Nanjing University. The ages of mice were 8–10 weeks and mice were maintained under 12 h light or dark cycles with unlimited access to food and water. All animal maintenance and experimental procedures were carried out in accordance with the US National Institute of Health Guidelines for Use of Experimental Animals and approved by the Institutional Animal Care and Use Committee of the Shanghai Jiaotong University School of Medicine, Shanghai, China.

Mice were anesthetized with 1% sodium pentobarbital (Westang biotechnology, Shanghai, China) by intraperitoneal injection (100 mg/kg). The dorsal hair was shaved and the skin was sterilized with betadine and alcohol. Full-thickness of skin defects of 1.5 × 1.5 cm^2^ were made on mice.

For observing the effect of autophagy during wound healing, the wounded C57BL/6 mice were divided into four groups (n = 48 per group,) as control and treated with rapamycin, 3-MA, and rapamycin plus 3-MA, respectively. Rapamycin (1 mg/kg/d) or 3-MA (10 mg/kg/d) was injected by intraperitoneal (i.p.) once per day, up to the wounds closed completely. Equal volume of PBS was used by i.p. in control group.

For diabetic wounds, full-thickness defects of 1.5 × 1.5 cm^2^ were created on db/db mice and the wounded db/db mice were divided into 3-MA treated and non-treated groups (n = 48 per group). The same size of skin defects were made on db/m mice as control (n = 48).

For AGEs related experiments, the wounded C57BL/6 mice were divided into three groups (n = 48 per group) as control, AGEs treatment, and AGEs plus 3-MA treatment. AGEs of 100 mg/kg were injected daily into margin tissues of wound.

The wounds images were collected using digital camera (Nikon J2, Tokyo, Japan) every day until the wounds closed completely. The morphometric analysis of the wounds was assessed using images of the wounds at days 0, 1, 3, 7, 10, 14, 17 and day 21 post-operatory to measure the remained wound area using Image J software (NIH, US). The rate of wound closure that represents the percentage of wound reduction from the original wound size was calculated using the following formula: wound area day 0 – wound area (day1, 3, 7, 10, 14, 17 and 21)/wound area day 0 × 100. Values expressed as percentage of the healed wounds. In addition, the tissue samples were harvested from the skin wounds at Days 0, 1, 3, 7, 10, 14 and 21 post-wounding, respectively, under anesthesia, for further usage.

### Macrophage culture and Polarization

The macrophage cell line of mice RAW264.7, was purchased from American Type Culture Collection (ATCC, Manassas, VA, US). Cells were cultured in DMEM supplemented with fetal bovine serum, penicillin and streptomycin. Cells were stimulated with LPS (100 ng/ml) and/or AGEs (100 μg/ml), or IL-4 (10 ng/ml) and/or AGEs for 24 h. To induce or inhibit autophagy, cells were treated with rapamycin (100 nM) or 3-MA (2 mM) plus CQ (50 nM) for 24 h (CQ was used for inhibiting autophagy flux).

### Immunohistochemical staining (IHC)

The skin tissues were fixed and embedded. Sections were made as a thickness of 4 μm. The procedure for IHC was followed as the instruction from antibodie manufactures. In briefly, the slides were deparaffinized, rehydrated, and blocked. Then, the slides were incubated with primary antibodies: anti-LC3 antibody (1:100), anti-Beclin1 antibody (1:100), and anti-AGEs antibody (1:500), respectively, overnight at 4 °C. After washing, the sections were added with secondary antibody (Boster, Wuhan, China) for 40 min at room temperature (RT). After washing, sections were incubated with an SABC solution according to the manufacturer’s instruction. Sections were observed under light microscopy (Nikon Ti-S, Tokyo, Japan). Staining were evaluated and scored by two independent researchers. The evaluation of staining results was semi-quantitatively scored for the extent of immunoreactions as follows: 0, 0% immunoreactive cells; 1, <5% immunoreactive cells; 2, 5–50% immunoreactive cells; 3, >50% immunoreactive cells. Additionally, the staining intensity was graded, including 0 (negative), 1 (weak), 2 (intermediate), or 3 (strong). The final immunoreaction score was defined as the sum of both parameters, and the samples were grouped as negative (0), weak (1–2), moderate (3), and strong (4–6) staining. The software Image J was used for intensity evaluation.

### Immunolabeling and confocal microscopy imaging

The sections of skin were processed as IHC procedure or RAW264.7 cells were fixed and blocked. Then slides were incubated with primary antibodies: anti-LC3 antibody (1:200), anti-F4/80 antibody (1:100), respectively, overnight at 4 °C. Alexa Fluor 488 conjugated goat anti-rabbit IgG second antibodies (1:1000) and/or Alexa Fluor 594 conjugated goat anti-rat IgG second antibodies (1:1000) were added on the specimens for 1 h at RT. In addition, the specimens were stained with 4′, 6-diamidino-2-phenylindole (DAPI) for visualizing nuclei. Images were taken under fluorescence microscope (Nikon Ti-S, Tokyo, Japan) or Laser Scanning Confocal Microscope (ZEISS LSM 710, Germany).

### Electron microscopy

The wounds specimens from mice or RAW264.7 cells were fixed in 2% glutaraldehyde and treated according to TEM instruction of the Shanghai Jiaotong University School of Medicine. Images were taken under electronic microscope (Philip Quanta-200, US).

### Patient tissue study

All samples were collected in No.3 hospital, Shanghai Jiaotong University School of Medicine from April 2013 to May 2015. Human tissue usage protocol in this study was approved by the ethics committee of No.3 hospital, Shanghai Jiaotong University School of Medicine (2013–022) and carried out in accordance with the requirements of regulations and procedures regarding to human subject protection laws such as GCP, written informed consent was obtained from all patients or their guardians for the use of the biospecimens for research purposes. The total of 74 skin tissue specimens were harvested from the chronic wounds patients with type 2 diabetic mellitus (n = 49, male 21 and female 23, range of ages 46–90 years old, average 55.4 ± 12.7) or without diabetes (n = 25, male 12 and female 13, range of ages 38–82 years old, average 52.2 ± 16.4). Normal skin tissue (unwounded) samples of 10 (out of 74) were collected from the diabetic patients (n=5) and non-diabetic patients (n = 5). Those tissues were discarded or redundant while a skin transplantation surgery was performed. All subjects have no history of self-immunological diseases. The specimens were subjected to IHC staining.

### Western blotting

Western blotting was employed for measuring LC3, S6K1 and pT389-S6K1 or IRF8 protein level. Cells were lysed with RIPA and PMSF. The protein concentration was evaluated by BCA protein assay kit. Equal amounts of protein were separated using 15% SDS-PAGE and then transferred to a polyvinylidene difluoride membranes (Millipore, US). The membranes were incubated with anti-LC3 antibody (1:2000), anti-S6K1 (1:1000), anti-pT389-S6K1 (1:1000), anti-IRF8 antibody (1:1000), or β-actin (1:2000)overnight at 4 °C. After washing, goat anti-rabbit IgG second antibody (1:5000) was added on the specimens for 1 h at RT. Protein bands were visualized using the enhanced chemiluminescence detection kit (Thermo scientific, Hudson, NH, US). Images were taken by fusion-capture software (Fusion FX7, France).

### Real-time polymerase chain reaction

Total RNA was isolated from RAW264.7 cells with Trizol reagent following the manufacturer’s protocol. Reverse transcription was carried out with 2 μg of RNA using a high capacity cDNA reverse transcription kit. Annealing of primers was done at 25 °C for 10 min, followed by elongation at 37 °C for 2 h and inactivation of the enzyme at 85 °C for 5 min. The primers are shown in Table 2 and were purchased from Sangon Biotech (Shanghai, China). PCR was performed using QuantiFast SYBR Green PCR Kit (Qiagen, Valencia, CA). Taq polymerase was activated at 95 °C for 5 min, the cycling parameters were denaturated at 95 °C for 30 sec and extension at 60 °C for 1 min (for 40 cycles). PCR was performed in triplicate in a Light Cycler 480 Real-Time PCR System (Roche, Swiss). Relative gene expressions were calculated using the 2-^ΔΔ^CT methods. Samples were normalized with *Gapdh* as the endogenous control gene.

### Flow cytometry

RAW264.7 cells were fixed with 10% buffered formalin solution, washed in PBS twice, and blocked with 2% BSA in PBS. Cells were incubated with specific monoclonal antibody (CD206-APC or CD11c-APC) diluted 1:50 in 2% BSA in PBS, for 15 min. Cells were analyzed using the FACSCalibur Flow Cytometer (BD Biosciences, San Jose, CA, US).

### RNA interference infection

shRNA oligonucleotide sequences for targeting mouse IRF8 gene mRNA was designed by Sunbio Medical Biotechnology (Shanghai, China). A scrambled sequence of targeting IRF8 sequence was used as a control, which was 5′-CCGGTTCTCCGAACGTGTCACGTTTCAAGAGAACGTGACACGTTCGGAGAATTTTTG-3′. The shIRF8 targeting sequence was 5′-CCGGGGACATTTCTGAGCCATATAACTCGAGATATGGCTCAGAAATGTCCAGTTTTTTG -3′. 293T cells were insfected by recombinant lentivirus with *irf8* or control. After 48 h, the supernatant was harvested, filtered and concentrated. To establish stable *irf8*-knockdown cell lines, RAW264.7 cells were transduced with lentiviral RNAi vector. The final rate of infection for RAW264.7 cells was 70% around, this was confirmed using real-time PCR or western blot.

### Statistical analysis

The data are presented using mean ± SD. The data were analyzed by Student’s t test. All analyses were performed using GraphPad Prism software (Version 5.0). Results were considered significant when *P* < 0.05.

## Additional Information

**How to cite this article**: Guo, Y. *et al*. AGEs Induced Autophagy Impairs Cutaneous Wound Healing via Stimulating Macrophage Polarization to M1 in Diabetes. *Sci. Rep.* **6**, 36416; doi: 10.1038/srep36416 (2016).

**Publisher’s note**: Springer Nature remains neutral with regard to jurisdictional claims in published maps and institutional affiliations.

## Figures and Tables

**Table 1:** IHC scores of LC3 in unwounded and chronic wounds skin from non-DM and DM patients.

	Unwounded Skin	Chronic Wounds
N-DM	0.60 ± 0.55 (n = 5)	4.55 ± 1.19 (n = 20)**
DM	1.00 ± 0.71 (n = 5)	5.36 ± 0.87 (n = 44)**^#^

**P < 0.01, compared to unwounded skin.

^#^*P* < 0.05, compared to non-DM groups.

Expressions of LC3 in chronic wounds from patients with or without diabetes were increased significantly than that in unwounded skin. Meanwhile, the levels of LC3 in chronic wounds from DM patients were higher than that from non-DM patients.

**Table 2:** Primer sequences for real-time PCR.

*Tnf-a*	Forward	5′-CCCTCACACTCAGATCATCTTCT-3′
	Reverse	5′-GCTACGACGTGGGCTACAG-3′
*Il1b*	Forward	5′-GCAACTGTTCCTGAACTCAACT-3′
	Reverse	5′-ATCTTTTGGGGTCCGTCAACT-3′
*Il6*	Forward	5′-CACATGTTCTCTGGGAAATCGTGGA-3′
	Reverse	5′- TCTCTCTGAAGGACTCTGGCTTTGT-3′
*Inos*	Forward	5′-GTTCTCAGCCCAACAATACAAGA-3′
	Reverse	5′-GTGGACGGGTCGATGTCAC-3′
*Arg-1*	Forward	5′-CTCCAAGCCAAAGTCCTTAGAG-3′
	Reverse	5′-AGGAGCTGTCATTAGGGACA-3′
*Il10*	Forward	5′-GCTCTTACTGACTGGCATGAG-3′
	Reverse	5′-CGCAGCTCTAGGAGCATGTG-3′
*Mrc-1*	Forward	5′-CTCTGTTCAGCTATTGGACGC-3′
	Reverse	5′-TGGCACTCCCAAACATAATTTGA-3′
*Mgl1*	Forward	5′-TGAGAAAGGCTTTAAGAACTGGG-3′
	Reverse	5′-GACCACCTGTAGTGATGTGGG-3′
*Gapdh*	Forward	5′-AGGTCGGTGTGAACGGATTTG-3′
	Reverse	5′-TGTAGACCATGTAGTTGAGGTCA-3′
