# Author Correction: AGEs Induced Autophagy Impairs Cutaneous Wound Healing via Stimulating Macrophage Polarization to M1 in Diabetes

**DOI:** 10.1038/s41598-023-49392-8

**Published:** 2024-01-16

**Authors:** Yuanyuan Guo, Cai Lin, Peng Xu, Shan Wu, Xiujun Fu, Weidong Xia, Min Yao

**Affiliations:** 1 Department of Burns and Plastic Surgery, Shanghai Ninth People’s Hospital, Institute of Traumatic Medicine, Shanghai Jiao Tong University School of Medicine, Shanghai, 201900 China; 2 Burn and Wound Center, First Affiliated Hospital of Wenzhou Medical University, Wenzhou, 325000 China; 3 Wellman Center for Photomedicine, Massachusetts General Hospital, Harvard Medical School, Boston, MA 02114 USA

Correction to: *Scientific Reports* 10.1038/srep36416, published online 02 November 2016

This original version of the article contains errors in Figs. 1 and 3.

In Fig. 1, as a result of an error in figure assembly, in Fig. 1F the images for a wrong condition were used. In addition, the images 7d, 14d, and 21d in Fig. 1F are identical to Fig. 3E, db/m, 7d, 14d, and 21d.

The corrected Figure 1 and accompanying legend appear below as Figure 1.

**Figure 1:**
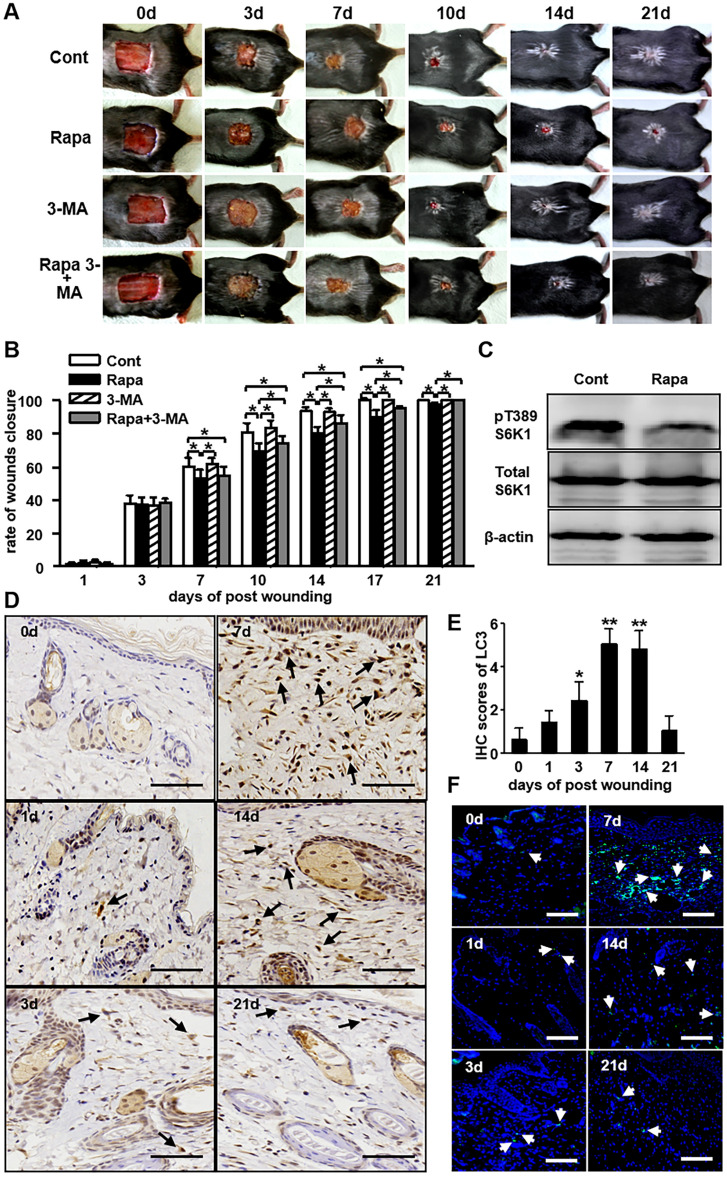
The role of autophagy in wound healing. (**a**) A full-thickness skin defect of 1.5 × 1.5 cm^2^ was made on C57BL/6 mouse. The mice were treated with rapamycin, 3-MA (10 mg/kg/d), rapamycin pluse 3-MA by i.p. Equal volume of PBS was used as control. Representative photographs of wounds closure are presented as indicated. (**b**) The areas of wounds were quantified and the ratios of wounds closure were expressed as a percentage of repaired wound compared to the area on day 0 (4 groups, n = 10 per group). Data is presented as the mean ± SD. *P < 0.05. (**c**) The levels of pT389-S6K1 in wounds with and without rapamycin (Rapa) treatment were detected by Western blot analysis on day 7 post-wounding. (**d**) Skin tissues from the control animals that wounded only were harvested for LC3 staining using IHC. Brown staining stands for the positive cells as indicated by black arrows. Scale bars = 100 μm. (**e**) The average scores of LC3 staining were evaluated using IHC (4 groups, 6 time points as indicated, n = 6 per group per time point). Data is presented as the mean ± SD. *P < 0.05, **P < 0.01. (**f**) Immunofluorescence was carried out with LC3 antibody in tissue from the control animals that wound only. White arrows indicate the positive cells with green staining. Blue staining represents nucleus. Scale bars = 100 μm.

Also in Fig. 3, as a result of an error in the figure assembly:

- Figure 3C, db/m 21d is partly overlapping with Fig. 2D, Cont 14d;
- Figure 3C, db/m 14d is partly overlapping with Fig. 2D, Cont 14d;
- Figure 3C, db/m 14d is partly overlapping with Fig. 3C, db/m 21d;
- Figure 3C, db/m 7d is partly overlapping with Fig. 5D, Cont 7d;
- Figure 3C, db/db 7d is partly overlapping with Fig. 5D, AGEs 7d.

**Figure 2:**
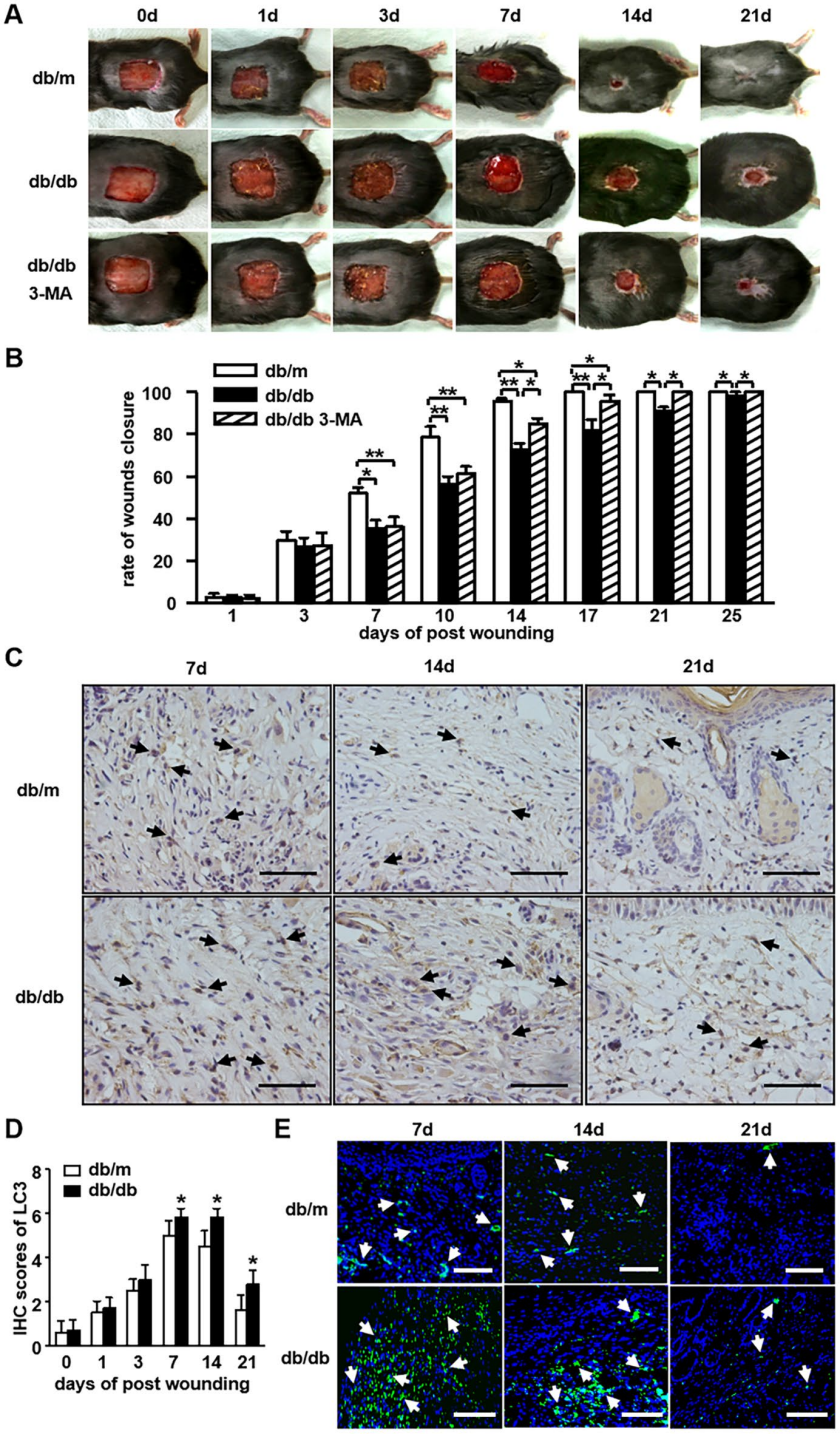
The changes of autophagy in impaired diabetic wounds healing. (**a**) A full-thickness skin defects was made on each db/db or db/m mice. The db/db mice were pre-treated with or without 3-MA (10 mg/kg/d) by i.p. from 1 day before wounding and up to the wounds closed completely. Representative photographs of wounds closure are presented. (**b**) The areas of wounds were quantified and the ratios of wounds closure were expressed (3 groups, n = 10 per group). Data is presented as the mean ± SD. *P < 0.05, **P < 0.01. (**c**) The LC3 staining was performed using IHC. The positive cells are brown as indicated by black arrows. LC3 positive cells in wounds in db/db mice are compared to that in db/m mice. Scale bars = 100 μm. (**d**) The average scores of LC3 staining were evaluated (two groups, 6 time points as indicated, n = 6 per group per time point). Data is presented as the mean ± SD. *P < 0.05. (**e**) Immunofluorescence was employed to determine LC3 positive expressions. Green staining represents the positive cells as indicated by white arrows, blue staining represents nucleus. Scale bars = 100 μm.

The corrected Fig. 3 and accompanying legend appear below as Fig. 2.

